# Unbalanced bidirectional causal association between thyroid cancer and ER-positive breast cancer: should we recommend screening for thyroid cancer in breast cancer patients?

**DOI:** 10.1186/s12864-023-09854-9

**Published:** 2023-12-11

**Authors:** Hongtao Wang, Shiwen Li, Jinyuan Shi, Chuyao Feng, Yanan Wang, Fan Zhang

**Affiliations:** 1 Department of Hematology, Shengjing Hospital of China Medical University, Shenyang, China; 2 Department of Thyroid Surgery, General Surgery, Qilu Hospital of Shandong University, Jinan, Shandong Province 250012 P. R. China; 3 Department of Endocrinology and Metabolism, Institute of Endocrinology, National Health Commission Key Laboratory of Diagnosis and Treatment of Thyroid Diseases, The First Hospital of China Medical University, Shenyang, Liaoning Province 110001 P. R. China

**Keywords:** Breast cancer, Causal, Mendelian, Randomization, Thyroid cancer

## Abstract

**Background:**

The association between breast cancer (BC) and thyroid cancer (TC) has been studied in several epidemiological studies. However, the underlying causal relationship between them is not yet clear.

**Methods:**

The data from the latest large-sample genome-wide association studies (GWAS) of BC and TC were searched in the public GWAS database. The BC GWAS data included estrogen receptor (ER)-positive and negative subgroups. Two-way two-sample Mendelian Randomization (MR) was used to explore the potential causal relationship between BC and TC. Inverse variance weighting (IVW) and the MR-Egger method were used to combine the estimation of each single nucleotide variation (previous single nucleotide polymorphism). BC was taken as the result, and the effect of TC exposure was analyzed. Then, the effect of BC exposure on the result of TC was analyzed.

**Results:**

Both IVW and MR-Egger results indicated that gene-driven thyroid cancer does not cause estrogen receptor-positive breast cancer and is a protective factor (β = -1.203, SE = 4.663*10^–4^, *P* = 0.010). However, gene-driven estrogen receptor-positive breast cancer can lead to the development of thyroid cancer (β = 0.516, SE = 0.220, *P* = 0.019).

**Conclusion:**

From the perspective of gene drive, people with TC are less likely to have ER-positive BC. In contrast, people with ER-positive BC are more likely to have TC. Therefore, it is recommended that patients with BC be screened regularly for TC.

**Supplementary Information:**

The online version contains supplementary material available at 10.1186/s12864-023-09854-9.

## Introduction

In recent years, with the advancement of various malignant tumor diagnoses and treatment methods, the survival rate of patients has increased significantly, and the incidence of secondary tumors caused by this has also increased. In the United States, secondary malignancies account for approximately 18% of all tumors [1]. In addition to common risk factors and sequelae of radiotherapy or chemotherapy, this result may also be caused by genetic susceptibility. Compared with the general population, the probability of another tumor developing into a secondary malignant tumor is significantly higher among patients with breast cancer (BC) and thyroid cancer (TC) after surgery or tumor carriers [2–9].

BC was the third most common incident cancer overall in 2007, with an estimated 2.0 million cases. The majority occurred in women and it caused 601,000 deaths in women and 11,000 deaths in men, making it the fifth leading cause of cancer deaths for both sexes combined in 2017 globally [10].

The incidence of TC continues to rise worldwide, and it has become the fifth most common malignant tumor for women in the United States. It is estimated that in 2015, there were more than 62,000 new cases in men and women [11]. It is well known that TC is gender-biased, with up to three times more diagnoses being made in the female population than the male population.

Many previous clinical studies of epidemiological and genetic evidence have proven the potential correlation between BC and TC [2–4, 12–19]. However, metachronous (occurring in succession, or called the precedence) BC and TC have not been identified clearly. The reasons may be due to a single factor or a combination of factors, such as genetic factors, environmental factors, and treatment-related factors [20]. For the current published literature, there is no evidence of a clear causal relationship between BC and TC. This is caused by the research type (most of the studies are retrospective) and the lack of studies with long-term follow-up and a large sample size.

Causal inference in traditional observational epidemiological research is hindered by the possibility of confounding and preserving causality [21]. Mendelian randomization (MR) research is a data analysis method that has been used in recent years in epidemiological etiology inference. It can be used to uncover causal relationships between an exposure and outcome in the presence of such limitations. It is a form of instrumental variable analysis in which genetic variation is used as an interest exposed proxy [22]. In this study, we used MR to explore the causal relationship between TC and BC.

## Materials and methods

### Data sources

The Integrative Epidemiology Unit Open genome-wide association studies (GWAS) Project website (https://gwas.mrcieu.ac.uk/) is a database of genetic associations from the GWAS summary datasets for researchers to query and download GWAS data. We searched for all BC-related GWAS on it and selected a GWAS meta-analysis conducted by Michailidou et al. [23], which was found to have the largest sample size of estrogen receptor (ER) positives or negatives. This study included the following: i. the Breast Cancer Association Consortium (BCAC) and Discovery, Biology and Risk of Inherited Variants in Breast Cancer Consortium (DRIVE); the subjects came from 68 collaborating studies (61,282 female BC cases and 45,494 female controls of European ancestry), ii. iSelect Collaborative Oncological Gene-Environment Study (iCOGS) project (46,785 cases and 42,892 controls), and iii. eleven other BC GWAS (14,910 cases and 17,588 controls). This study included a total of 122,977 BC and 105,974 controls of European ancestry, which included 10,680,257 single-nucleotide polymorphisms (SNPs, GWAS ID: ieu-a-1126). These data included the subgroup GWAS data of ER (+) (GWAS ID: ieu-a-1127) and ER (-) (GWAS ID: ieu-a-1128). The sample sizes were 175,475 and 127,442, respectively.

Similar to the GWAS databases for BC, the GWAS data of TC was also found on the same website. The single study by Kohler et al. [24] was conducted as a GWAS in an Italian population of 690 TC cases and 497 controls (sample size was 1,187). This study contained a total of 572,028 SNPs (GWAS ID: ieu-a-1082).

### Genetic variants used as instruments

MR utilizes genetic variants as instrumental variables (IVs), which are associated with an outcome only through their association with a particular risk factor (for example, TC) [22]. MR relies upon three assumptions: first, that the IV is associated with the risk factor of interest (*p* < *5* × *10*^*–*8^); second, that the IV is not affected by the confounding factors acting upon the association between the risk factor and outcome of interest; and finally, that the IV is associated with the outcome of interest only via its effect on the modifiable risk factor. A conservative Bonferroni correction adjusted for the number of exposures and primary outcomes analyzed in the study was applied to control for false-positive findings due to multiple testings. A *P*-value less than 0.05 (/No.SNPs) was considered statistically significant in all analyses. A *P*-value less than 0.05 was considered as evidence for nominal significance.

Based on the results of the currently largest GWAS on TC and BC, we identified 328, 62, 47, and 19 independent (*r*^2^ ≤ 0.01 within windows of ± 1 Mb for variants in the same locus) IVs associated at a genome-wide significant level with TC, overall BC, ER(+), and ER(-), respectively. Of note, quality-controlled IVs based on a minor allelic frequency (MAF) ≥ 0.05 were selected.

The analysis process was performed between TC and the overall BC and ER(+), ER(-) BC bidirectionally.

### Mendelian randomization estimation

A bidirectional, two-sample MR was also performed. We used the Cochrane Q test and MR-Egger method to evaluate heterogeneity and horizontal pleiotropy. Here, known genetic variants for our predictor traits of interest were extracted and two different methods of two-sample MR were performed. Firstly, in the inverse variant weighted (IVW) instrumental variable analysis, IVW assumed that all genetic instruments were valid, and therefore, susceptible to horizontal pleiotropy whereby variants had an effect on the outcome via a route other than the risk factor of interest. To reduce this potential source of bias, we also used the MR-Egger techniques that are more robust to pleiotropy [25, 26]. In the MR-Egger analysis, the intercept was unconstrained to remove the assumption that all variants were valid instrumental variables and allowed a weighted regression. This reduced the possibility of variants having a stronger effect on the outcome than the exposure trait. It is worth noting that if the results of the heterogeneity test indicated significant heterogeneity between IVs, then the random-effects model of IVW was referred to. If the heterogeneity test results indicated insufficient heterogeneity, then the regression results of the fixed effects model needed to be referred to.

### Statistical analysis

Statistical analysis was performed using R version 4.0.3 (https://www.r-project.org/), R package “TwoSampleMR” version 0.5.6 (https://github.com/MRCIEU/TwoSampleMR) and “devtools” version 2.4.0 (https://www.rdocumentation.org/packages/devtools/).

The MR analysis in the present study was carried out mainly from the following two aspects: to respectively discuss the influence of gene-driven TC on BC, and the influence of gene-driven BC on TC.

## Results

### Characteristics of the included IVs

Detailed information on each IV is listed in Supplementary Tables 1 and 2, which is respectively based on the MR analysis on BC, ER(+), ER(-), and TC. The correlation power of each IV was assessed by the f statistic (= β_exposure_^2^/SE_exposure_^2^). As shown in Supplementary Tables 1 and 2, the minimum values of the f statistics were 29.6197 and 29.7366. All genetic risk scores associated strongly with their corresponding traits, with all f statistics greater than 10.

### Bidirectional MR between TC and BC

**Fig. 1:**
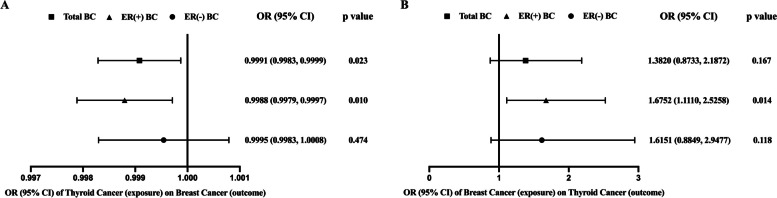
**A** The relationship between TC and BC with TC as the exposure factor and BC as the outcome factor. **B** The relationship between TC and BC with BC as the exposure factor and TC as the outcome factor

As shown in Table 1, TC was associated with BC (Beta = -9.204*10^–4^, SE = 4.046*10^–4^, *p* = 0.023), indicating that TC is a significant protective factor for total BC. Additionally, TC was also associated with ER(+) BC (Beta = -1.203*10^–3^, SE = 4.663*10^–4^, *p* = 0.010), indicating that TC also is a significant protective factor for ER(+) BC. However, significant results were not found for ER(-) BC (Beta = -4.559*10^–4^, SE = 6.368*10^–4^, *p* = 0.474). Therefore, the protective role of TC is especially profound for ER(+) BC (Fig. 1A).

**Table 1:** Results of MR between genetically predicted thyroid cancer (exposure, *N* = 1187) and breast cancer (outcome, *N* = 228,951), ER (+) breast cancer (outcome, *N* = 175,475), ER (-) breast cancer (outcome, *N* = 127,442)

Outcome	Analysis type	NSNP	Beta	SE	*p* value	Q	Q-*p* value	Egger intercept	Intercept-*p* value
Breast Cancer	IVW (Random)	328	-9.204 × 10^–4^	4.046 × 10^–4^	0.023	567.222	3.683 × 10^–15^	-4.123 × 10^–4^	0.638
	IVW (Fixed)	328	-9.204 × 10^–4^	3.072 × 10^–4^	0.003	567.222	3.683 × 10^–15^		
	MR-Egger	328	-6.477 × 10^–4^	7.059 × 10^–4^	0.360	566.835	3.022 × 10^–15^		
ER (+)	IVW (Random)	328	-1.203 × 10^–3^	4.663 × 10^–4^	0.010	529.97	7.990 × 10^–12^	-1.116 × 10^–3^	0.268
	IVW (Fixed)	328	-1.203 × 10^–3^	3.663 × 10^–4^	0.001	529.97	7.990 × 10^–12^		
	MR-Egger	328	-4.656 × 10^–4^	8.124 × 10^–4^	0.567	527.979	9.218 × 10^–12^		
ER (-)	IVW (Random)	328	-4.559 × 10^–4^	6.368 × 10^–4^	0.474	426.033	1.825 × 10^–4^	-3.149 × 10^–5^	0.982
	IVW (Fixed)	328	-4.559 × 10^–4^	5.579 × 10^–4^	0.414	426.033	1.825 × 10^–4^		
	MR-Egger	328	-4.351 × 10^–4^	1.112 × 10^–3^	0.696	426.032	1.581 × 10^–4^		

Q and Q-*p* value represent the Cochran’s Q value and corresponding *p* value for estimated heterogeneity; Egger intercept and intercept-*p* value represent estimated pleiotropy effect and corresponding *p* value

*IVW* Inverse variant weighted

On the contrary, in Table 2 of the relationship between BC and TC, total and ER(-) BC was not associated with TC (*p* = 0.167 and *p* = 0.118), indicating that ER(-) BC has no causal association with TC. However, ER(+) BC was associated with TC (Beta = 0.516, SE = 0.210, *p* = 0.014), indicating that ER(+) BC is a significant risk factor for TC (Fig. 1B).

**Table 2:** Results of MR between genetically predicted breast cancer (exposure, *N* = 228,951), ER (+) breast cancer (exposure, *N* = 175,475), ER (-) breast cancer (exposure, *N* = 127,442) and thyroid cancer (outcome, *N* = 1187)

Exposure	Analysis type	NSNP	Beta	SE	*p* value	Q	Q-*p* value	Egger intercept	Intercept-*p* value
Breast Cancer	IVW (Random)	62	0.324	0.234	0.167	86.118	0.019	-0.037	0.045
	IVW (Fixed)	62	0.324	0.197	0.101	86.118	0.019		
	MR-Egger	62	0.908	0.761	0.237	85.192	0.018		
ER (+)	IVW (Random)	47	0.516	0.220	0.019	50.724	0.293	0.014	0.050
	IVW (Fixed)	47	0.516	0.210	0.014	50.724	0.293		
	MR-Egger	47	0.306	0.764	0.691	50.632	0.261		
ER (-)	IVW (Random)	19	0.479	0.307	0.118	39.917	2.142 × 10^–3^	0.084	0.089
	IVW (Fixed)	19	0.479	0.206	0.020	39.917	2.142 × 10^–3^		
	MR-Egger	19	-0.255	0.837	0.764	37.930	2.512 × 10^–3^		

Q and Q-*p* value represent the Cochran’s Q value and corresponding *p* value for estimated heterogeneity; Egger intercept and intercept-*p* value represent estimated pleiotropy effect and corresponding *p* value

*IVW* Inverse variant weighted

## Discussion

There have been many studies that have reported a possible link between BC and TC, but it is difficult to determine whether there is indeed a link between BC and TC, and in which direction. Nielsen et al. conducted a meta-analysis that confirmed and quantified the increased likelihood of the co-existence of BC and differentiated thyroid cancer [27]. Due to the inevitable heterogeneity in the meta-analysis study that originated from the included literature, and that the included literature was after the discovery of one kind of tumor, Nielsen et al. could not quantify the time between the first tumor and the second tumor. They found that the risk of two tumors becoming each other’s secondary tumors increased, indicating that control bias alone cannot fully explain this connection, and there may be potential pathophysiological risks. Therefore, in order to further clarify whether there is a potential causal relationship between these two cancers, a new analytical research method called Mendelian randomization opened up new ideas for our research.

The Mendelian law of inheritance stipulates those alleles obtained from parents are passed to offspring through random separation. Therefore, it is unlikely that the genotype of the offspring is related to the confounding factors in the population. In addition, when the fertilized egg is formed, the germline genotype of the offspring is already fixed, which precedes the observed variable in time, avoiding the problem of reverse causality. The MR method involves looking for genetic variants associated with exposure and then testing the association between these variants and the results. When the necessary conditions are met, the causal “disambiguation” relationship between exposure and outcome can be estimated [28].

In recent years, a variety of MR research methods have been summarized, all of which use genetic variation to infer the causal relationship between features of interest, such as two-sample MR, two-step MR, multivariable MR, and factorial MR [29, 30]. Bidirectional MR is one of those above in which the tools of exposure and outcome are used to assess whether the “exposure” variable causes the “outcome”, or whether the “outcome” variable causes the “exposure” [31]. First of all, “exposure” and “outcome” variables need to be defined. The MR analysis is first carried out from “exposure” to “outcome”, and then in the opposite direction (i.e., from “outcome” to “exposure”). MR analysis is performed by using SNPs that are robustly related to each trait in the individual GWAS. The principle is to assume that the causal relationship between two variables works through the potential mechanism, and MR analysis can determine the direction of the causal sequence that acts in the mechanism [30]. Therefore, MR promises to be a valuable strategy to examine causality in complex biological/omics networks for disease prevention in the future.

Similar to breast tissue, both benign and malignant thyroid tissue are highly responsive to circulating estrogen [32]. In fact, elevated circulating hormone levels are associated with TC [33, 34]. Hyperestrogenism (elevated endogenous estrogen) during reproductive years is associated with an increased prevalence of TC in women of reproductive age; however, HRT (Hormone replacement therapy) or other exogenous estrogen exposure are not linked to TC [34–36]. Additionally, estrogen may serve as a link between the co-occurrence of autoimmune thyroid disorders and BC, which predominantly affect women [37]. Immune tolerance and the development of autoimmunity is largely controlled by the AIRE gene [38], estrogen is a key regulator of this gene, and reduced activity from elevated estrogen contributes to autoimmune susceptibility [38–42]. Overall, sex hormones, primarily estrogen have a connection to not only BC, but also thyroid malignancy.

In our study, MR analysis based on a large sample size GWAS study revealed a potential causal relationship between BC and TC; that is, TC may be a protective factor for BC occurrence (especially in ER-positive BC), and ER-positive BC is a risk factor for TC occurrence. Since subgroup analysis based on whether ER is positive had produced different results, we believe that ER has a potential pathophysiological role in the occurrence, development, and secondary tumors of breast and TC. However, ER itself is one of the main molecular targets in BC pathogenesis and is expressed in approximately 70% of invasive BC. It is a steroid hormone receptor and a transcription factor that, when activated by estrogen, activates oncogenic growth pathways in BC cells. Therefore, using endocrine agents to downregulate ER signaling is the primary systemic therapy for ER-positive BCs [43]. On the aspect of TC, estrogen plays its growth-promoting role through classical genomic and non-genomic pathways mediated by membrane-bound ER [44]. It is also a potent growth factor both for benign and malignant thyroid cells, which may explain the sex difference in the prevalence of TC [45]. Therefore, ER-positive may play an important role between BC and TC.

This study has the following advantages and limitations. First, we chose the largest GWAS database on BC and TC to ensure that our results are true and reliable. Secondly, we used MR for the first time to conclude that genetic prediction found that TC is a protective factor for BC, and BC is a risk factor for TC. This also provides a clear explanation for some previous epidemiological investigations and provides a basis for TC screening for BC patients. Thirdly, this study is the first to perform subgroup analysis by estrogen receptor, detailing the relationship between BC and TC in the subgroup above mentioned. However, the GWAS data in this study are all derived from European populations. Therefore, it is not clear whether the results of this study are applicable to populations or races in other regions. In addition, the data of BC patients in this study uses only whether ER is positive for subgroup analysis. The lack of progesterone receptor-positive data (and triple-negative BC) makes it impossible to conduct a more accurate subgroup analysis of BC patients. We believe that if more sample sizes and pathological types of GWAS data can be included in the future, the results of MR will be more directional and provide a clear direction for basic research to explore the potential relationships.

## Conclusion

This study used a bidirectional two-sample MR approach to explore the potential causal relationships between BC and TC. It was found that patients with TC are less likely to have ER-positive BC; on the contrary, people with ER-positive BC are more likely to have TC. It is recommended that patients with BC be screened regularly for TC.

### Supplementary Information

**Additional file 1: Supplementary Table 1.** Detailed information of the selected SNPs between Thyroid cancer (exposure, *N*=1187) and Breast cancer (Outcome 1, *N*=228,951), ER (+) Breast cancer (Outcome 2, *N*=175,475), ER (-) Breast cancer (Outcome 3, *N*=127,442). **Supplementary Table 2.** Detailed information of the selected SNPs between Breast cancer (exposure, *N*=228,951), ER (+) Breast cancer (exposure, *N*=175,475), ER (-) Breast cancer (exposure, *N*=127,442) and Thyroid cancer (Outcome, *N*=1187).

## Data Availability

All data were from the Integrative Epidemiology Unit Open genome-wide association studies (GWAS) Project website (https://gwas.mrcieu.ac.uk/). The original contributions statement presented in the study are included in the article/Material. Further inquiries can be directed to the corresponding authors.

## Abbreviations

BC: Breast cancer
TC: Thyroid cancer
GWAS: Genome-wide association studies
ER: Estrogen receptor
MR: Mendelian randomization
BCAC: Breast Cancer Association Consortium
DRIVE: Discovery, Biology and Risk of Inherited Variants in Breast Cancer Consortium
iCOGS: ISelect Collaborative Oncological Gene-Environment Study
SNPs: Single-nucleotide polymorphisms
IVs: Instrumental variables
MAF: Minor allelic frequency
IVW: Inverse variance weighting
