# Supplementary material for: Unbalanced bidirectional causal association between thyroid cancer and ER-positive breast cancer: should we recommend screening for thyroid cancer in breast cancer patients?
Source: BMC Genomics. 2023 Dec 11;24:762. doi: 10.1186/s12864-023-09854-9 (PMC10712093; doi:10.1186/s12864-023-09854-9)
Supplement: Supplementary file 1 — Additional file 1: Supplementary Table 1. Detailed information of the selected SNPs between Thyroid cancer (exposure, N=1187) and Breast cancer (Outcome 1, N=228,951), ER (+) Breast cancer (Outcome 2, N=175,475), ER (-) Breast cancer (Outcome 3, N=127,442). Supplementary Table 2. Detailed information of the selected SNPs between Breast cancer (exposure, N=228,951), ER (+) Breast cancer (exposure, N=175,475), ER (-) Breast cancer (exposure, N=127,442) and Thyroid cancer (Outcome, N=1187). [file 12864_2023_9854_MOESM1_ESM.pdf]

Supplementary Table 1. Detailed information of the selected SNPs between Thyroid cancer (exposure, N=1187) and Breast cancer (Outcome 1, N=228951), ER (+) Breast cancer (Outcome 2, N=175475), ER (-) Breast cancer (Outcome 3, N=127442).

SNPs	Effect allele	Other allele	Chr	β for exposure	SE for exposure	p value for exposure	β for outcome 1	SE for outcome 1	p value for outcome 1	β for outcome 2	SE for outcome 2	p value for outcome 2	β for outcome 3	SE for outcome 3	p value for outcome 3
rs1001948	C	A	10	2.08667	0.1023	1.37E-104	0.00E+00	0.0071	0.9949	-8.00E-04	0.0085	0.9223	0.0046	0.0129	0.72
rs10031777	C	T	4	1.20899	0.08867	9.30E-44	0.0243	0.0062	8.35E-05	0.023	0.0074	0.001795	0.0045	0.0113	0.69
rs10049939	G	A	4	3.0297	0.1997	1.39E-86	-0.009	0.0071	0.2063	-0.0131	0.0085	0.1226	-0.0055	0.013	0.67
rs10124357	C	T	9	2.05416	0.1069	1.43E-94	0.0082	0.0064	0.2	0.0161	0.0076	0.03427	-0.009	0.0116	0.43
rs1019024	C	T	8	1.29088	0.09109	2.02E-47	0.0115	0.0076	0.1287	0.0108	0.009	0.2325	0.0156	0.0136	0.25
rs10199888	T	C	2	-1.204	0.09614	1.42E-37	-0.0116	0.0091	0.2002	-0.0114	0.0108	0.2917	-0.02	0.0164	0.22
rs10208038	G	T	2	0.54043	0.08517	1.93E-10	-0.0064	0.0066	0.3314	-0.0031	0.0078	0.688201	-0.0216	0.0118	0.07
rs1021224	C	A	5	0.62594	0.08892	1.50E-12	-0.0014	0.0076	0.8527	-0.0065	0.009	0.4742	0.0073	0.0136	0.59
rs10260654	T	G	7	-0.521	0.08762	2.42E-09	0.0002	0.0065	0.979	0.0013	0.0077	0.8617	-0.0106	0.0118	0.37
rs1028400	C	A	1	0.56002	0.08672	8.95E-11	0.0209	0.0066	0.001454	0.014	0.0078	0.073729	0.0178	0.0119	0.13
rs10416371	C	A	19	0.54116	0.08627	3.06E-10	-0.0009	0.0068	0.8976	-0.005	0.0082	0.5419	0.0015	0.0126	0.91
rs10474588	C	A	5	0.66359	0.08487	3.95E-15	0.0108	0.0065	0.0957	0.0033	0.0077	0.666201	0.0189	0.0118	0.11
rs10475268	T	G	5	-0.5741	0.08545	1.55E-11	0.0109	0.0067	0.1036	0.0213	0.0079	0.007132	-0.004	0.0121	0.74
rs10493096	A	C	1	-0.6832	0.117	3.40E-09	0.0074	0.0085	0.3853	-0.0067	0.0101	0.5056	0.0235	0.0153	0.13
rs10494448	C	A	1	-0.5522	0.09228	1.83E-09	0.0076	0.0064	0.2343	0.0096	0.0077	0.2123	0.0135	0.0116	0.25
rs10502692	T	C	18	-3.8903	0.4175	1.82E-54	0.0076	0.008	0.3448	0.0072	0.0095	0.453401	-0.0012	0.0145	0.94
rs10513889	C	A	18	0.46825	0.08528	3.69E-08	-2.60E-03	0.0062	0.673101	-3.00E-04	0.0074	0.964	0.0041	0.0113	0.72
rs10762573	C	A	10	0.7683	0.09634	7.78E-16	0.0127	0.0066	0.0558	0.0207	0.0079	0.009054	5.00E-04	0.0122	0.97
rs1077420	C	A	19	-2.0691	0.1107	2.70E-90	0.0018	0.0063	0.7709	0.002	0.0075	0.7871	0.0041	0.0115	0.72
rs10779770	C	A	1	0.70058	0.1254	1.48E-08	-0.0141	0.0094	0.1354	-0.0077	0.0112	0.496	-0.0323	0.0169	0.06
rs10786634	G	T	10	0.57323	0.08545	1.67E-11	-0.0052	0.0064	0.4164	-0.0076	0.0076	0.3171	-0.0058	0.0116	0.62
rs10787889	T	G	10	-0.6509	0.08824	1.20E-13	-0.0036	0.0071	0.6086	-0.003	0.0084	0.7188	0.0086	0.0127	0.5
rs10797423	C	A	1	1.97472	0.09458	4.14E-105	-0.0004	0.008	0.9613	0.0012	0.0095	0.8978	7.00E-04	0.0144	0.96
rs10812736	A	C	9	-0.7631	0.1119	4.65E-12	-0.0242	0.0081	0.002882	-0.0211	0.0097	0.02928	-0.0318	0.0147	0.03
rs1082023	T	G	7	-1.9618	0.101	1.93E-93	-0.0015	0.0067	0.8227	0.0011	0.008	0.8875	-0.0082	0.0121	0.5
rs10832835	C	A	11	-2.3086	0.1175	1.54E-104	0.0096	0.0066	0.1441	0.0139	0.0079	0.07761	0.0139	0.012	0.25

rs10833197	A	C	11	-0.5795	0.08526	9.00E-12	0.0119	0.0065	0.06765	0.0086	0.0078	0.2707	0.0298	0.0118	0.01
rs10869031	G	T	9	0.55356	0.08603	1.06E-10	0.0041	0.0063	0.5141	0.0041	0.0075	0.580801	0.0055	0.0114	0.63
rs10894838	T	G	11	-2.4588	0.1547	1.43E-77	0.0111	0.0078	0.1559	0.0072	0.0094	0.4461	0.0046	0.0142	0.75
rs10916258	A	C	1	-0.735	0.1172	2.07E-10	0.0185	0.0078	0.01717	0.0199	0.0093	0.03145	0.0395	0.0142	0.01
rs10928200	T	G	2	-0.7619	0.09678	1.84E-15	-0.0063	0.0067	0.346	-0.0106	0.008	0.183	0.0037	0.0121	0.76
rs10949938	T	G	7	2.06593	0.1197	2.65E-79	-0.0038	0.0068	0.5823	0	0.0081	0.9964	-0.0144	0.0123	0.24
rs10952871	T	G	7	-0.4938	0.0879	1.75E-08	0.0036	0.0064	0.580399	0.013	0.0077	0.09165	-0.0154	0.0117	0.19
rs10954267	A	C	7	-0.6763	0.08515	1.42E-15	0.0024	0.0069	0.7264	0.0097	0.0083	0.2452	-0.0225	0.0127	0.08
rs11006981	C	A	10	-2.3654	0.2435	5.01E-32	-0.0213	0.0093	0.02206	-0.0169	0.0111	0.1293	-0.0321	0.0171	0.06
rs11036050	A	C	11	-0.5153	0.08632	2.09E-09	0.0003	0.0068	0.9604	0.0044	0.0081	0.5882	0.0018	0.0122	0.88
rs11041733	T	G	11	-2.0988	0.09586	5.06E-117	-3.70E-03	0.007	0.5971	-9.00E-04	0.0084	0.9137	0.012	0.0127	0.34
rs11098432	C	A	4	0.49348	0.08449	4.68E-09	0.0086	0.0063	0.1691	0.0067	0.0075	0.3682	-0.0012	0.0114	0.92
rs11132917	G	T	4	0.556	0.0875	1.77E-10	-0.0007	0.0069	0.9186	0.0065	0.0082	0.4327	0.0076	0.0125	0.54
rs1123570	A	C	5	-1.9031	0.111	4.54E-75	-0.0056	0.007	0.4214	-0.0064	0.0084	0.4432	-0.0106	0.0126	0.4
rs1154205	G	T	18	0.51998	0.0856	1.09E-09	-0.049	0.0063	9.49E-15	-0.0618	0.0076	2.88E-16	-0.0057	0.0115	0.62
rs11592120	T	G	10	-0.5632	0.08591	4.68E-11	-0.0028	0.0064	0.664099	-0.0117	0.0077	0.1253	-0.0083	0.0117	0.48
rs11638590	C	A	15	-0.6678	0.08758	1.80E-14	0.0011	0.0067	0.8695	0.0018	0.008	0.8182	0.0053	0.0122	0.66
rs11688848	A	C	2	-0.7952	0.1122	6.33E-13	-0.0002	0.0079	0.9805	-0.0016	0.0095	0.8653	-0.0068	0.0144	0.64
rs11692610	C	T	2	-2.469	0.1757	4.02E-63	0.0039	0.0073	0.5951	-0.0041	0.0087	0.6353	0.0054	0.0132	0.68
rs11703587	C	A	22	2.17068	0.09776	4.20E-121	-0.007	0.0069	0.3114	-0.0082	0.0083	0.3233	0.009	0.0126	0.48
rs11708894	T	G	3	-1.8171	0.09591	8.23E-87	-0.0013	0.0071	0.8497	0.0045	0.0084	0.589	-0.0139	0.0127	0.27
rs11721807	T	G	4	-0.7158	0.09962	3.94E-13	-0.0011	0.0068	0.8764	-0.0093	0.0081	0.2541	0.0082	0.0124	0.51
rs11743743	A	C	5	-0.7265	0.08588	1.74E-17	-0.0098	0.0063	0.1208	-0.0117	0.0075	0.1203	-0.0089	0.0115	0.44
rs11786147	G	T	8	0.51998	0.0856	1.11E-09	-0.0033	0.0063	0.600701	-0.0033	0.0075	0.664899	-0.0034	0.0115	0.77
rs11883054	G	T	19	0.80923	0.09519	8.53E-18	-0.0118	0.0078	0.1298	-0.0127	0.0094	0.1792	-0.0247	0.0141	0.08
rs11927465	G	T	3	0.61471	0.08493	3.65E-13	2.30E-03	0.0066	0.725	-1.00E-04	0.0079	0.9932	0.0129	0.012	0.28
rs1193787	T	C	11	-0.6655	0.08469	2.82E-15	0.0039	0.0064	0.5414	0.0018	0.0077	0.8189	0.0245	0.0119	0.04
rs11975969	G	T	7	2.13962	0.1062	2.15E-103	-2.70E-03	0.0063	0.670101	5.00E-04	0.0075	0.9446	-0.0056	0.0114	0.62
rs11993947	G	T	8	0.57436	0.08591	1.93E-11	-0.012	0.0063	0.05696	-0.016	0.0075	0.03239	-0.0169	0.0115	0.14
rs12040716	C	A	1	0.52886	0.08897	2.44E-09	-0.0019	0.0071	0.793299	0.0077	0.0085	0.3651	-0.0146	0.0129	0.26
rs12118017	C	A	1	0.66166	0.09973	2.23E-11	0.0074	0.0075	0.3209	0.0132	0.0089	0.1404	-0.006	0.0137	0.66
rs12140722	C	A	1	0.63552	0.08543	7.86E-14	-0.0071	0.0065	0.2762	-0.008	0.0078	0.3038	-0.0097	0.0118	0.41

rs12151689	C	A	2	0.66146	0.09328	9.14E-13	0.0112	0.0065	0.086551	0.0063	0.0078	0.416	0.029	0.0119	0.02
rs1216502	C	A	11	0.59258	0.1073	2.57E-08	-0.0125	0.0087	0.1504	-0.014	0.0105	0.1825	0.0018	0.0157	0.91
rs12186611	A	C	5	-0.6062	0.09393	8.42E-11	0.0154	0.0067	0.02254	0.0242	0.008	0.002584	0.0165	0.0122	0.17
rs12196235	C	A	6	2.06436	0.09556	3.04E-113	-0.0085	0.0073	0.2422	-0.0163	0.0087	0.0612	-0.0062	0.0132	0.64
rs12210929	A	C	6	-0.5317	0.08478	3.15E-10	0.0054	0.0066	0.4171	0.0061	0.0079	0.4425	0.0162	0.012	0.17
rs1224751	T	C	6	-0.6136	0.08499	4.14E-13	0.007	0.0066	0.2861	0.0109	0.0079	0.1654	0.0097	0.012	0.42
rs12403117	A	C	1	-0.538	0.09403	8.96E-09	5.50E-03	0.0068	0.4172	5.00E-04	0.0081	0.9497	0.0158	0.0124	0.2
rs12433740	T	G	14	-0.6692	0.09594	2.05E-12	-0.0003	0.0065	0.9615	0.0017	0.0077	0.8233	-0.0116	0.0118	0.33
rs12441088	T	G	15	-1.2146	0.09031	1.11E-42	0.0041	0.0073	0.5723	0.0061	0.0086	0.4787	-0.003	0.0132	0.82
rs12469609	G	T	2	0.68142	0.08627	2.02E-15	-0.0019	0.0063	0.764901	0.0015	0.0075	0.8464	-0.0092	0.0115	0.42
rs12510224	G	T	4	-2.1663	0.1379	1.41E-69	0.0005	0.0068	0.944	-0.0058	0.0081	0.473	-0.0026	0.0122	0.83
rs12524738	A	G	6	-2.7216	0.319	1.08E-28	-0.02	0.0109	0.06656	-0.0162	0.013	0.2131	-0.0042	0.0198	0.83
rs12564912	T	G	1	-2.4284	0.1237	1.92E-107	0.0066	0.0066	0.3204	0.0049	0.0079	0.5384	0.0024	0.012	0.84
rs12595914	T	G	16	-0.5635	0.08528	3.32E-11	0.0057	0.0066	0.3887	0.009	0.0079	0.2542	0.0027	0.012	0.82
rs12651136	A	C	4	-2.0114	0.1169	1.12E-77	-5.60E-03	0.0063	0.3734	8.00E-04	0.0074	0.914	-0.0182	0.0115	0.11
rs12715419	T	G	3	-0.5793	0.08599	1.35E-11	0.0477	0.0063	2.71E-14	0.0492	0.0075	4.69E-11	0.0223	0.0114	0.05
rs12782349	A	C	10	-0.5556	0.09054	6.99E-10	0.0011	0.0066	0.8655	0.005	0.0079	0.5282	-0.0035	0.012	0.77
rs12908044	A	C	15	2.13369	0.1224	6.63E-82	-0.0036	0.0081	0.6576	-0.0019	0.0099	0.8456	-0.021	0.0146	0.15
rs12925224	G	T	16	0.62474	0.08909	1.78E-12	0.0034	0.0069	0.6248	-0.0047	0.0082	0.5701	0.009	0.0125	0.47
rs13019891	T	G	2	-2.0159	0.1114	7.99E-85	-2.00E-04	0.0062	0.9778	-3.00E-04	0.0075	0.9727	-0.0072	0.0114	0.53
rs1321947	A	C	20	-0.5172	0.08434	7.73E-10	-0.0055	0.0069	0.4267	-0.0057	0.0083	0.490801	-0.0189	0.0125	0.13
rs1325201	T	G	1	0.6362	0.08591	1.00E-13	-0.0102	0.0062	0.097681	-0.0053	0.0073	0.471	-0.0233	0.0113	0.04
rs1327144	C	A	1	0.71393	0.08551	4.51E-17	0.0101	0.0063	0.1108	0.0084	0.0076	0.2676	0.0276	0.0115	0.02
rs13292880	A	C	9	-1.8971	0.1078	2.64E-78	0.0086	0.0063	0.1708	0.0141	0.0075	0.06117	0.0028	0.0115	0.8
rs13404338	G	A	2	2.13453	0.1038	1.14E-106	-0.0072	0.0065	0.271	-0.01	0.0077	0.1934	0.0038	0.0119	0.75
rs1358852	C	A	6	0.5318	0.0902	3.16E-09	0.0016	0.0074	0.8245	0.0104	0.0089	0.2435	-0.0148	0.0135	0.27
rs1365242	C	A	15	2.20003	0.1105	1.42E-103	0.0129	0.0066	0.052309	0.0115	0.0079	0.1449	0.0354	0.0121	0
rs1370301	G	T	4	0.6436	0.08547	3.89E-14	0.0006	0.0062	0.9241	0.0039	0.0074	0.6002	0.0047	0.0113	0.68
rs1388492	T	C	3	0.53018	0.08976	3.00E-09	-0.0066	0.0074	0.371	-0.0055	0.0088	0.5329	-0.0156	0.0133	0.24
rs1390241	C	A	3	0.5449	0.08565	1.72E-10	0.0013	0.0064	0.8345	-0.0019	0.0076	0.8065	0.0093	0.0116	0.42
rs1401186	A	C	11	-0.5289	0.08457	3.48E-10	0.0002	0.0062	0.9787	-0.0033	0.0074	0.6524	-0.0035	0.0113	0.76
rs1414154	A	C	13	-0.4631	0.08447	3.81E-08	0.0033	0.0064	0.6041	0.0046	0.0077	0.5508	-0.0092	0.0117	0.43

rs1442664	T	G	18	-1.9207	0.09942	1.08E-91	0.0119	0.0071	0.096159	0.0113	0.0086	0.1868	0.0096	0.0129	0.45
rs1459522	T	G	3	-0.5236	0.08729	1.75E-09	-0.0042	0.0063	0.500299	0.0066	0.0075	0.3754	-0.0159	0.0115	0.16
rs1467345	A	G	7	-0.8829	0.1109	5.21E-16	0.0113	0.0101	0.2624	0.0084	0.012	0.4839	0.0166	0.0181	0.36
rs1468696	G	T	3	0.50203	0.08463	2.72E-09	0.0081	0.0063	0.2019	0.0059	0.0076	0.4365	0.0249	0.0114	0.03
rs1481897	A	C	8	-0.5437	0.09031	1.48E-09	-0.0016	0.0064	0.8026	0.0046	0.0076	0.5408	-3.00E-04	0.0116	0.98
rs1482176	T	G	3	-0.6259	0.08637	3.33E-13	0.0132	0.0063	0.03783	0.0091	0.0076	0.227	0.0167	0.0115	0.15
rs1499214	T	G	3	-0.604	0.09358	8.33E-11	0.0077	0.0071	0.2779	0.0054	0.0085	0.5284	0.0256	0.0129	0.05
rs150929	T	G	16	-0.4818	0.08677	2.58E-08	0.0019	0.0068	0.785301	-0.0043	0.0081	0.5961	0.027	0.0123	0.03
rs1511954	T	G	15	-0.6607	0.08938	1.03E-13	-0.0092	0.0066	0.1638	-0.0059	0.0079	0.4557	-0.0246	0.0121	0.04
rs153709	A	C	5	-0.5466	0.08647	2.25E-10	-0.0009	0.0064	0.8862	-0.007	0.0076	0.3551	-0.004	0.0116	0.73
rs1547039	A	C	14	-1.9929	0.0943	7.24E-108	0.0056	0.0077	0.4679	0.009	0.0093	0.3327	-0.0122	0.0139	0.38
rs1552706	T	G	8	0.56194	0.09269	1.10E-09	-0.0096	0.007	0.1723	-0.0085	0.0084	0.313	-0.029	0.0126	0.02
rs156166	C	A	3	0.60605	0.08453	6.06E-13	0.0031	0.007	0.6567	0.0034	0.0083	0.686099	0.0091	0.0126	0.47
rs1562058	A	C	4	-0.4803	0.08818	4.68E-08	-0.008	0.0063	0.2068	-0.0058	0.0075	0.4456	-0.0089	0.0115	0.44
rs1577026	A	C	13	-2.4688	0.2104	2.66E-46	-3.00E-03	0.0084	0.7218	-2.00E-04	0.01	0.9816	-0.0168	0.0153	0.27
rs1594467	G	A	6	2.62073	0.1073	6.18E-156	0.0002	0.007	0.9769	-0.0029	0.0084	0.725	0.0147	0.0126	0.25
rs1610315	A	C	6	-2.0786	0.0962	1.63E-113	-0.0078	0.0071	0.2693	-0.0065	0.0084	0.439	-0.0111	0.013	0.39
rs163192	A	C	5	-0.5683	0.08593	3.20E-11	-0.0017	0.0065	0.7975	-0.0016	0.0078	0.8391	0.0024	0.0118	0.84
rs163952	C	A	5	2.44369	0.1274	2.07E-104	-0.0059	0.0065	0.3573	-0.0069	0.0077	0.3705	0.006	0.0117	0.61
rs1667822	T	G	3	-1.9704	0.09571	1.92E-102	-0.0053	0.0069	0.4435	-0.0068	0.0082	0.4063	-0.0171	0.0125	0.17
rs1683564	A	C	19	-0.6392	0.09313	4.87E-12	0.0094	0.0078	0.2294	0.0073	0.0095	0.4418	0.025	0.014	0.07
rs16838428	T	C	4	-2.9546	0.2916	2.19E-42	0.009	0.0099	0.3636	0.0077	0.0117	0.5139	0.0273	0.0182	0.13
rs17018881	A	C	3	-0.7482	0.1219	4.72E-10	0.0004	0.0084	0.9624	-0.0075	0.01	0.453799	0.0211	0.0151	0.16
rs17039540	A	C	4	-0.5812	0.1068	4.15E-08	-0.0011	0.0069	0.8714	0.0013	0.0082	0.8788	-0.0159	0.0126	0.2
rs17137948	A	C	7	0.6669	0.08455	2.25E-15	0.0053	0.0068	0.4323	0.0089	0.0081	0.2723	0.0019	0.0122	0.88
rs17256058	T	G	14	-0.6925	0.1255	2.26E-08	-4.20E-03	0.0096	0.6655	8.00E-04	0.0115	0.9443	-0.0084	0.0178	0.63
rs17294252	A	C	4	-0.6453	0.1159	1.81E-08	-0.0088	0.0074	0.2318	-0.0113	0.0088	0.1995	-0.0189	0.0135	0.16
rs1750330	C	A	1	0.46474	0.08413	3.07E-08	-0.0009	0.0063	0.8828	-0.0013	0.0075	0.8614	-0.0043	0.0114	0.7
rs17571067	A	C	1	-0.5695	0.09178	4.44E-10	-0.0095	0.0065	0.1432	-0.0083	0.0077	0.2831	-0.0052	0.0118	0.66
rs17600706	T	G	14	-0.5708	0.09982	8.72E-09	0.007	0.0071	0.3289	0.0062	0.0085	0.4668	0.022	0.013	0.09
rs17647119	A	C	18	-0.5656	0.1007	1.57E-08	0.0064	0.0076	0.3945	0.0012	0.009	0.894	0.0158	0.0137	0.25
rs1792746	A	C	18	-0.6234	0.08744	7.84E-13	-0.0024	0.0068	0.7294	-0.001	0.0082	0.9028	0.0039	0.0123	0.75

rs1813617	A	C	2	-0.5108	0.0875	4.64E-09	-0.0045	0.0064	0.481599	0.003	0.0076	0.6951	-0.0141	0.0116	0.23
rs1822338	C	A	11	0.52004	0.08711	2.10E-09	-0.0057	0.0067	0.3929	-0.009	0.008	0.2617	-3.00E-04	0.0121	0.98
rs1827488	G	T	15	0.58179	0.08911	5.44E-11	0.0076	0.0066	0.2502	0.0059	0.0079	0.4601	0.0011	0.012	0.93
rs1846861	C	A	2	0.65277	0.08532	1.51E-14	-0.0213	0.0065	0.001163	-0.0253	0.0078	0.001224	-0.0118	0.0118	0.32
rs1860990	T	G	9	-0.6743	0.1038	5.51E-11	0.0147	0.0077	0.05464	0.0078	0.0092	0.397	0.0123	0.0138	0.37
rs1868534	G	T	11	0.52071	0.08434	5.93E-10	0.0006	0.0064	0.926	0.0049	0.0076	0.5205	-0.0012	0.0116	0.92
rs1880256	C	A	2	0.63281	0.0972	5.46E-11	-0.0027	0.0067	0.6834	-0.0101	0.008	0.2043	0.0044	0.0122	0.72
rs1885597	G	T	14	0.4984	0.08517	4.42E-09	-0.0025	0.0063	0.690799	-0.0023	0.0075	0.7554	0.0095	0.0114	0.4
rs1887387	T	C	9	-0.6419	0.08793	2.15E-13	-1.60E-03	0.0078	0.8419	4.00E-04	0.0094	0.9686	-0.0063	0.0141	0.66
rs1889562	G	T	6	0.62861	0.08707	3.94E-13	0.0033	0.0071	0.64	0.0118	0.0085	0.1637	-0.0095	0.0129	0.46
rs1898422	A	C	2	-0.6878	0.1108	3.50E-10	1.50E-03	0.0085	0.8625	9.00E-04	0.0101	0.9265	-0.0022	0.0153	0.88
rs1971100	T	G	9	-0.7608	0.1186	7.52E-11	-0.0001	0.0087	0.992	-0.0071	0.0104	0.4935	0.0222	0.0159	0.16
rs197559	G	T	21	0.49397	0.08473	5.03E-09	3.60E-03	0.0066	0.5838	-8.00E-04	0.0079	0.9176	0.0143	0.012	0.23
rs1983033	A	C	15	-0.596	0.09325	1.29E-10	-0.0101	0.0068	0.1385	-0.013	0.0082	0.1121	-0.0238	0.0124	0.05
rs2001823	T	G	2	-0.5944	0.08694	6.47E-12	-0.0115	0.008	0.1482	-0.012	0.0095	0.2087	-0.0098	0.0145	0.5
rs2011347	G	T	2	0.62755	0.08816	8.29E-13	-0.0001	0.0062	0.9838	0.0023	0.0074	0.760599	-0.0145	0.0113	0.2
rs2026912	A	C	10	-1.0815	0.1473	2.78E-14	0.0009	0.0083	0.9102	-0.0089	0.0099	0.3699	0.0131	0.015	0.38
rs2044241	A	C	5	-0.6233	0.09468	3.46E-11	0.0177	0.0074	0.01724	0.0165	0.0089	0.06253	0.0229	0.0135	0.09
rs2121697	C	A	2	-0.5309	0.08663	7.77E-10	-0.0019	0.0062	0.765399	0.0025	0.0074	0.732	0.0074	0.0114	0.52
rs2156354	G	T	18	2.01066	0.09555	1.57E-107	-0.0009	0.0076	0.9102	0.0025	0.009	0.780301	-0.0232	0.0138	0.09
rs2157787	C	A	16	2.05885	0.09758	1.30E-108	0.0041	0.0069	0.5459	-0.0011	0.0082	0.8942	0.0092	0.0125	0.46
rs2184234	G	T	6	0.61267	0.08597	8.27E-13	-0.0039	0.0062	0.5254	-0.0103	0.0074	0.1654	0.0078	0.0113	0.49
rs219254	G	A	7	2.76208	0.1871	1.76E-75	-0.0038	0.0069	0.5858	-0.0056	0.0083	0.4986	0.0019	0.0126	0.88
rs2197176	C	A	2	0.6298	0.1106	9.15E-09	-0.0022	0.0084	0.7901	-0.0013	0.01	0.8955	0.0087	0.0152	0.57
rs2236674	A	C	21	-0.6307	0.08952	1.39E-12	0.0139	0.0078	0.077011	0.0102	0.0094	0.2773	-0.0061	0.014	0.66
rs2239214	G	A	14	1.55878	0.1133	1.55E-47	0.0008	0.0119	0.9481	0.0045	0.0144	0.756	-0.0198	0.0218	0.36
rs2243497	C	A	13	0.53086	0.08709	9.62E-10	-0.0067	0.0065	0.3004	-0.0173	0.0077	0.02449	-3.00E-04	0.0118	0.98
rs2250057	A	C	9	-1.3486	0.09026	1.63E-52	-0.0049	0.0066	0.4551	-0.0077	0.0078	0.3256	-0.0101	0.0119	0.39
rs2292324	G	C	16	-4.6511	0.7135	3.28E-42	0.0101	0.0107	0.3438	0.0171	0.0126	0.1769	-0.0243	0.0195	0.21
rs2336409	C	A	8	0.58612	0.08502	4.60E-12	-0.0044	0.0064	0.4925	-0.0019	0.0076	0.8079	-0.0116	0.0116	0.32
rs2368105	T	G	2	-0.5497	0.08459	7.03E-11	0.0145	0.0067	0.02908	0.0098	0.008	0.2195	0.0199	0.0121	0.1
rs2414003	T	G	15	-0.7402	0.1139	4.42E-11	-0.0011	0.007	0.8695	-0.0073	0.0083	0.3797	0.0273	0.0126	0.03

rs2452477	T	G	2	-0.6646	0.119	1.60E-08	-0.0006	0.0086	0.9486	0.0127	0.0103	0.2175	-0.012	0.0158	0.45
rs2483374	C	A	13	0.68241	0.08626	1.80E-15	-0.0054	0.0063	0.3924	-0.0098	0.0075	0.193	-0.0132	0.0115	0.25
rs2484667	C	A	10	0.47375	0.08544	2.65E-08	-0.0004	0.0063	0.9465	0.001	0.0076	0.892	-0.0154	0.0116	0.19
rs2488100	G	T	6	0.5295	0.08505	4.20E-10	0.0032	0.0065	0.627001	0.004	0.0078	0.6059	7.00E-04	0.0119	0.95
rs2566511	T	C	7	-2.4953	0.1819	3.38E-61	0.0036	0.0076	0.639401	0.003	0.009	0.7374	0.0015	0.0138	0.91
rs2567501	C	A	17	2.18214	0.09786	9.31E-122	-3.50E-03	0.0069	0.611699	-4.00E-04	0.0082	0.9605	-0.0084	0.0126	0.5
rs2589810	A	C	5	-0.5371	0.08446	1.82E-10	-0.0136	0.0063	0.03135	-0.016	0.0075	0.03303	-0.001	0.0115	0.93
rs2640734	T	C	8	-2.2173	0.1503	1.35E-63	0.0108	0.0074	0.1472	0.013	0.0089	0.1426	-0.0045	0.0135	0.74
rs2677879	T	G	18	-1.4762	0.0908	2.95E-62	0.001	0.0066	0.875	0.0035	0.0079	0.655101	0.001	0.0119	0.94
rs2679163	T	G	2	-2.0723	0.1175	3.54E-82	-0.0091	0.0067	0.1747	-0.0082	0.008	0.3007	-0.0023	0.0121	0.85
rs26887	G	A	5	1.93167	0.1541	3.24E-44	0.0093	0.0187	0.6202	0.0117	0.0221	0.5975	-0.0584	0.0342	0.09
rs2690106	T	G	6	-0.5931	0.0871	7.96E-12	-0.0077	0.0063	0.2272	-0.0102	0.0076	0.1775	0.0085	0.0115	0.46
rs2720392	A	C	7	-0.6343	0.08724	2.72E-13	-0.0184	0.0063	0.003646	-0.0138	0.0075	0.067189	-0.0338	0.0115	0
rs2722633	C	A	3	0.60367	0.08513	1.07E-12	-0.004	0.0065	0.5374	-0.0063	0.0077	0.4106	-0.0036	0.0118	0.76
rs272620	G	T	5	0.53298	0.08958	2.30E-09	0.0046	0.0067	0.4951	0.0018	0.008	0.8224	-0.0018	0.0123	0.88
rs274012	A	C	7	-0.488	0.08535	9.94E-09	0.0047	0.0072	0.51	0.0087	0.0086	0.3094	0.0134	0.013	0.31
rs2756315	G	T	6	0.63243	0.08548	1.07E-13	-0.0049	0.0064	0.4439	-0.003	0.0076	0.694201	0.001	0.0117	0.93
rs2802523	C	A	13	1.98268	0.09573	1.07E-103	-0.0016	0.0068	0.8097	-0.0087	0.0081	0.2871	-0.0014	0.0123	0.91
rs2836836	T	G	21	-2.2118	0.1152	1.50E-97	0.0028	0.0068	0.6829	0.0042	0.0081	0.6072	-0.0048	0.0122	0.7
rs28433203	T	G	1	-2.721	0.2341	1.28E-49	0.0107	0.0073	0.1399	0.0053	0.0087	0.542	0.0164	0.0133	0.22
rs2844353	G	T	3	0.56531	0.08457	2.00E-11	0.017	0.0067	0.01116	0.0214	0.008	0.007512	0.0061	0.0122	0.62
rs2866442	G	T	4	1.83822	0.103	5.43E-79	-0.0044	0.0063	0.484	-0.0044	0.0075	0.5559	-0.0127	0.0115	0.27
rs2867803	A	C	7	-0.4814	0.08448	1.11E-08	0.0073	0.0063	0.2433	0.0035	0.0075	0.6373	0.0052	0.0114	0.65
rs2868166	A	C	7	-2.6709	0.1417	1.64E-108	0.0151	0.0068	0.02659	0.0045	0.0081	0.5756	0.0445	0.0123	0
rs2870132	A	C	20	-0.5841	0.1066	3.40E-08	-0.0013	0.0076	0.8655	0.0013	0.009	0.8838	0.0034	0.0137	0.8
rs2911738	G	T	8	-0.6358	0.08945	8.82E-13	0.0022	0.0065	0.7334	0.0015	0.0077	0.8454	-0.0071	0.0117	0.55
rs2973786	T	G	5	-2.0425	0.09622	3.33E-109	0.0091	0.0069	0.1892	0.0134	0.0082	0.1033	0.0096	0.0125	0.45
rs2981073	C	A	8	0.53883	0.08884	1.12E-09	-5.70E-03	0.0066	0.3904	-4.00E-04	0.0079	0.9598	0.0054	0.0121	0.65
rs30958	G	T	16	0.5394	0.09706	2.32E-08	-0.0165	0.0084	0.05074	-0.0166	0.0101	0.1013	-0.0036	0.015	0.81
rs3101528	G	T	8	2.07545	0.1128	4.04E-88	0.0083	0.0062	0.1781	0.0186	0.0074	0.01173	-0.0059	0.0113	0.6
rs3594	A	C	8	-0.6442	0.08906	3.47E-13	0.0073	0.0067	0.2767	0.0157	0.008	0.05029	-0.0046	0.0123	0.71
rs3745720	C	A	19	0.61748	0.08453	2.19E-13	-0.0077	0.0066	0.2373	-0.0034	0.0078	0.666699	-0.0065	0.0119	0.58

rs3769754	C	A	2	0.59056	0.0851	3.24E-12	4.90E-03	0.0067	0.4596	5.00E-04	0.008	0.9478	0.0143	0.012	0.23
rs3813636	G	T	1	0.69816	0.08497	1.44E-16	0.0043	0.0064	0.5054	0.006	0.0076	0.4342	0.0042	0.0116	0.72
rs3829647	G	T	19	0.49102	0.08798	2.16E-08	-1.70E-03	0.007	0.8127	-6.00E-04	0.0083	0.9418	0.0036	0.0129	0.78
rs3844222	G	T	16	1.35363	0.0896	1.36E-53	-0.0012	0.007	0.866	-0.0012	0.0084	0.8871	0.0039	0.0127	0.76
rs4143816	T	G	10	1.974	0.1095	2.93E-83	0.0035	0.0062	0.5716	0.0058	0.0074	0.4331	-0.0062	0.0113	0.58
rs4148124	C	A	21	0.70098	0.08845	1.50E-15	0.0016	0.007	0.8168	0.0021	0.0083	0.7988	-0.0128	0.0125	0.31
rs4233904	C	A	2	0.62605	0.09423	2.25E-11	-0.0273	0.0066	3.16E-05	-0.0283	0.0078	0.000299	-0.0205	0.012	0.09
rs4255455	C	A	10	0.63111	0.08543	1.17E-13	-0.0083	0.007	0.2345	-0.0057	0.0084	0.499001	0.0053	0.0125	0.68
rs4269220	G	T	4	0.48646	0.0857	1.25E-08	0.0173	0.0064	0.007201	0.016	0.0077	0.03691	0.0353	0.0117	0
rs4332846	A	C	19	-0.5833	0.08519	6.21E-12	-0.0103	0.007	0.1403	-0.0138	0.0083	0.095691	-0.001	0.0125	0.94
rs4336409	T	G	5	-0.5005	0.08621	5.77E-09	-2.80E-03	0.0063	0.656999	6.00E-04	0.0075	0.9403	-0.0079	0.0114	0.49
rs4358768	T	G	8	-2.2917	0.1344	1.49E-82	-0.0119	0.007	0.0869	-0.011	0.0083	0.186	-0.0013	0.0126	0.92
rs4391665	G	A	1	2.3758	0.1392	3.23E-85	0.0033	0.0065	0.61	0.0109	0.0077	0.1558	0.0096	0.0117	0.41
rs4548277	T	G	9	2.30409	0.1487	4.85E-71	0.0021	0.0067	0.7516	0.0066	0.008	0.4049	-8.00E-04	0.0122	0.95
rs4564540	T	G	15	-2.0948	0.1049	9.57E-101	-0.005	0.0063	0.4255	-0.0108	0.0075	0.1515	-0.0104	0.0115	0.37
rs4567782	C	A	17	0.54801	0.08616	1.73E-10	9.00E-03	0.0062	0.1445	-9.00E-04	0.0074	0.9059	0.0289	0.0113	0.01
rs461599	C	A	5	0.51919	0.08459	7.49E-10	0.0055	0.0062	0.3801	0.007	0.0074	0.3459	0.0207	0.0114	0.07
rs4648935	T	G	1	-0.69	0.08918	7.02E-15	-0.0099	0.0062	0.1097	-0.0111	0.0074	0.135	-0.0077	0.0113	0.49
rs4658359	T	G	1	2.26433	0.1609	1.40E-59	-0.0051	0.0074	0.4913	-0.0031	0.0088	0.7298	-0.0145	0.0134	0.28
rs4679695	C	A	3	0.55984	0.085	3.83E-11	-0.0034	0.0064	0.595701	-0.0046	0.0077	0.5489	-0.0129	0.0117	0.27
rs4709487	T	G	6	-0.755	0.1259	1.11E-09	-0.0007	0.008	0.9302	-0.0014	0.0095	0.8792	0.0081	0.0146	0.58
rs4721443	G	T	7	0.68577	0.1056	5.40E-11	3.20E-03	0.0078	0.684499	-6.00E-04	0.0093	0.9505	-0.0079	0.014	0.57
rs4738050	G	T	8	0.51903	0.08615	1.49E-09	-0.0039	0.0062	0.5364	-0.0074	0.0074	0.3205	-2.00E-04	0.0113	0.99
rs4792390	A	C	17	-0.777	0.09028	3.96E-18	-0.0026	0.0072	0.720299	0.0021	0.0086	0.8035	-0.0056	0.0129	0.66
rs4808027	G	T	19	0.61465	0.1043	2.88E-09	-0.0033	0.0086	0.6979	-0.0106	0.0103	0.303	0.0189	0.0156	0.22
rs4867811	G	T	5	0.55389	0.09123	1.07E-09	-0.0097	0.0073	0.1801	-0.0159	0.0086	0.06635	-0.0035	0.0133	0.79
rs4881077	A	C	10	-1.9973	0.09473	2.11E-107	6.10E-03	0.0085	0.477	7.00E-04	0.0103	0.9446	0.0027	0.0156	0.86
rs4882110	T	G	11	-0.6004	0.08493	1.27E-12	0.0034	0.0062	0.5852	0.008	0.0074	0.2805	-0.0043	0.0114	0.7
rs4884522	A	C	13	0.69455	0.08585	4.20E-16	-0.0056	0.0065	0.3837	-0.0121	0.0077	0.1168	0.0023	0.0119	0.84
rs4886485	C	A	15	0.51103	0.08451	1.30E-09	0.0077	0.0065	0.2366	0.0188	0.0077	0.0152	-4.00E-04	0.0118	0.97
rs4896219	A	C	6	-0.5409	0.08547	2.13E-10	-0.0116	0.007	0.098299	-0.0086	0.0084	0.3061	-0.0201	0.0126	0.11
rs4904753	T	G	14	-0.6132	0.09848	3.58E-10	-0.0112	0.0066	0.08831	-0.0058	0.0078	0.4558	-0.0216	0.0121	0.08

rs4922081	A	C	8	-2.0859	0.1254	8.21E-75	0.0038	0.0066	0.5642	0.0039	0.0078	0.6155	0.0027	0.0119	0.82
rs4961718	C	A	9	1.92552	0.1027	8.88E-88	-0.0047	0.0063	0.4622	-0.0098	0.0076	0.1962	-0.0039	0.0115	0.73
rs4962362	C	A	10	-2.0948	0.1193	6.53E-82	-0.0016	0.0081	0.8421	0.0024	0.0097	0.807	-0.0134	0.0145	0.35
rs513154	G	T	3	0.60679	0.08845	5.37E-12	0.00E+00	0.0066	0.9951	1.00E-04	0.0079	0.9915	-0.0023	0.012	0.85
rs546583	C	A	1	0.563	0.08451	2.30E-11	0.0035	0.0066	0.596901	0.0021	0.0079	0.7896	0.0041	0.012	0.73
rs550201	C	A	18	-1.9944	0.1013	4.39E-96	0.0009	0.0067	0.8936	0	0.008	0.9978	0.0077	0.0121	0.52
rs5749581	A	C	22	-0.6173	0.08496	2.90E-13	-0.0031	0.0067	0.642	0.0048	0.008	0.5488	0.0011	0.0121	0.92
rs576070	T	G	5	-2.0174	0.1127	2.07E-83	0.0112	0.0064	0.080251	0.019	0.0076	0.01256	0.0145	0.0116	0.21
rs6046115	T	G	20	-0.6712	0.08508	2.24E-15	-0.0004	0.0067	0.9558	-0.0012	0.008	0.876	0.0052	0.0121	0.67
rs6054459	T	G	20	-1.9235	0.1053	1.22E-83	0.016	0.0065	0.01375	0.013	0.0077	0.09301	0.0172	0.0117	0.14
rs6060124	A	C	20	-0.7565	0.1134	1.36E-11	0.0156	0.007	0.02564	0.0145	0.0084	0.084101	0.0171	0.0129	0.18
rs630573	C	A	11	0.60422	0.09198	3.96E-11	0.005	0.0063	0.4288	0.0111	0.0075	0.1369	0.0039	0.0114	0.73
rs630695	G	T	6	-0.7702	0.1228	1.87E-10	0.011	0.0079	0.1622	0.007	0.0094	0.4593	-0.0072	0.0144	0.62
rs638335	T	G	1	-1.4014	0.1176	1.09E-35	-0.0166	0.0102	0.1026	-0.0245	0.0121	0.04239	-0.0255	0.0184	0.17
rs641603	T	G	15	-2.2462	0.1057	1.53E-115	-0.0033	0.0065	0.6136	-0.0045	0.0077	0.5571	-0.0065	0.0118	0.58
rs6430168	G	T	2	0.62642	0.09948	2.22E-10	-0.007	0.0079	0.3752	-0.0067	0.0094	0.475	-0.007	0.0142	0.62
rs6440877	A	C	3	-0.5014	0.08547	4.08E-09	-5.50E-03	0.0064	0.3871	4.00E-04	0.0076	0.9534	7.00E-04	0.0116	0.95
rs6471521	C	A	8	0.53597	0.08988	2.12E-09	-0.0023	0.0063	0.720701	-0.0068	0.0075	0.3642	-0.0063	0.0115	0.58
rs6478287	G	T	9	2.05338	0.09882	3.80E-106	0.0005	0.0064	0.9404	-0.0105	0.0077	0.1705	0.0104	0.0117	0.37
rs6498272	T	G	16	0.6044	0.09119	2.67E-11	0.0084	0.0066	0.2032	0.0119	0.0079	0.1322	0.011	0.012	0.36
rs6505863	C	A	18	0.71335	0.09359	1.52E-14	-0.0011	0.0063	0.8624	-0.0056	0.0075	0.4579	0.0017	0.0115	0.88
rs6516819	G	T	21	0.58609	0.08564	6.35E-12	-0.0109	0.0063	0.08459	-0.0101	0.0076	0.1805	-0.0249	0.0115	0.03
rs6532146	A	C	4	-2.1371	0.1116	2.71E-95	-0.0054	0.0062	0.3848	0.0014	0.0074	0.8519	-0.0156	0.0113	0.17
rs6533109	C	A	4	-0.4929	0.0858	8.32E-09	0.0113	0.0064	0.07625	0.0164	0.0076	0.03104	0.0178	0.0116	0.13
rs6540082	T	G	16	-0.491	0.08694	1.45E-08	-0.0014	0.0069	0.8432	0.0039	0.0082	0.6338	-0.0058	0.0126	0.65
rs6546667	G	A	2	0.69415	0.08459	1.59E-16	-0.0035	0.0064	0.586001	-0.0037	0.0076	0.6233	0.0141	0.0116	0.22
rs6565631	C	A	17	0.60112	0.09994	1.39E-09	1.00E-04	0.0081	0.9855	6.00E-04	0.0096	0.949	0.0045	0.0144	0.76
rs6578080	T	G	8	-0.5642	0.08475	2.37E-11	-0.0107	0.0066	0.1032	-0.0132	0.0079	0.09288	-0.0161	0.0119	0.18
rs6584869	C	A	10	0.64037	0.09282	3.82E-12	-0.0023	0.0068	0.732501	-0.0013	0.0081	0.8751	-0.0261	0.0123	0.03
rs662393	G	T	11	2.33904	0.159	1.21E-65	-0.0196	0.0067	0.003606	-0.0182	0.008	0.02307	-0.0339	0.0123	0.01
rs6670601	C	A	1	0.47433	0.08456	1.86E-08	0.0009	0.0063	0.8908	-0.0052	0.0075	0.4913	0.0036	0.0115	0.76
rs678476	A	C	1	-0.6379	0.09004	1.02E-12	-0.0002	0.0067	0.9727	-0.0013	0.008	0.873	-0.0164	0.0124	0.19

rs6793262	G	T	3	1.35712	0.08969	7.57E-54	0.0023	0.0062	0.7106	-0.0085	0.0074	0.2507	0.0291	0.0113	0.01
rs6827297	G	T	4	0.72155	0.09454	1.38E-14	-4.00E-04	0.0073	0.952	-2.00E-04	0.0088	0.9831	0.0078	0.0132	0.56
rs6828245	A	C	4	-0.6954	0.08646	6.04E-16	0.0074	0.0062	0.238	0.0102	0.0074	0.1721	0.0173	0.0113	0.13
rs6870385	C	A	5	0.50154	0.08994	2.17E-08	-0.0065	0.0065	0.3188	-0.0042	0.0078	0.5872	-0.0028	0.0119	0.81
rs6882903	C	A	5	0.54111	0.09258	4.30E-09	0.0078	0.007	0.2666	0.0053	0.0085	0.5299	-0.0069	0.0127	0.59
rs6919522	A	C	6	-0.7038	0.09155	9.43E-15	-0.0137	0.0068	0.04525	-0.0081	0.0082	0.3227	-0.0211	0.0125	0.09
rs6937429	G	T	6	0.53137	0.08824	1.49E-09	-0.0148	0.0066	0.02486	-0.0098	0.0079	0.2142	-0.0268	0.012	0.03
rs6944364	G	T	7	-0.5496	0.08559	1.17E-10	0.0029	0.0066	0.6556	-0.0012	0.0078	0.882	0.0045	0.0119	0.7
rs6946868	C	A	7	-2.0174	0.1086	3.51E-88	0.0077	0.0064	0.2277	0.0081	0.0077	0.2965	0.027	0.0116	0.02
rs6949292	A	C	7	-0.5373	0.09206	4.55E-09	-0.0021	0.0063	0.743401	-0.0113	0.0075	0.1326	0.0108	0.0115	0.35
rs6951443	A	C	7	-0.5283	0.08707	1.14E-09	0.0006	0.0063	0.9185	-0.0074	0.0075	0.3226	0.0155	0.0115	0.18
rs698171	C	A	5	1.91122	0.09431	6.96E-99	0.0062	0.0072	0.3842	0.0082	0.0085	0.3355	-0.0033	0.013	0.8
rs6984781	G	T	8	0.62273	0.09803	1.59E-10	0.0107	0.008	0.1794	0.0167	0.0095	0.078821	-0.0136	0.0146	0.35
rs7034893	C	T	9	-1.9303	0.1346	4.67E-56	0.0097	0.0068	0.1509	0.0081	0.0081	0.3169	0.0069	0.0123	0.57
rs711943	C	A	3	0.46172	0.08438	4.14E-08	-0.0031	0.0064	0.6295	-0.0058	0.0077	0.4481	-0.0028	0.0117	0.81
rs7186875	C	A	16	0.57839	0.09559	1.16E-09	-1.00E-03	0.0073	0.8914	-3.00E-04	0.0088	0.97	0.0021	0.0134	0.87
rs7188594	A	C	16	-0.5635	0.09117	5.25E-10	-0.0085	0.0073	0.2443	-0.0044	0.0087	0.6109	-0.0219	0.0131	0.09
rs7234149	C	A	18	1.91596	0.108	1.03E-79	-0.0079	0.0063	0.2078	-0.0011	0.0075	0.8817	-0.014	0.0115	0.22
rs7271235	G	T	20	1.99805	0.1049	1.52E-91	-0.0017	0.0068	0.7974	0.0054	0.0081	0.5043	-0.0174	0.0122	0.15
rs738667	A	C	22	-2.1893	0.09913	9.84E-121	0.0001	0.0065	0.989	0.0038	0.0078	0.6285	0.0109	0.0119	0.36
rs7516666	T	G	1	-2.0055	0.09526	4.70E-107	0.016	0.0071	0.02369	0.019	0.0085	0.02518	0.0098	0.0129	0.45
rs7528076	A	C	1	-0.5683	0.08718	5.92E-11	0.018	0.0067	0.006842	0.0187	0.0079	0.01832	0.0242	0.0121	0.04
rs754038	C	A	10	1.29755	0.08914	1.05E-49	-0.0015	0.0068	0.8253	-0.0041	0.0081	0.6153	0.0116	0.0123	0.35
rs7623609	T	G	3	0.49217	0.08971	3.67E-08	0.0081	0.0062	0.1934	0.0145	0.0074	0.051171	-0.0032	0.0114	0.78
rs7735978	A	C	5	-2.5653	0.1992	5.06E-56	0.0095	0.0079	0.2297	0.0216	0.0095	0.02234	0.0072	0.0144	0.62
rs7770868	A	C	6	-0.6182	0.08499	2.79E-13	0.0005	0.0064	0.9387	0.0073	0.0076	0.3369	-0.0168	0.0116	0.15
rs7781839	A	C	7	-0.4892	0.0858	1.06E-08	-0.0115	0.0066	0.08175	-0.0093	0.0079	0.2399	0.004	0.012	0.74
rs7815102	G	T	8	0.5032	0.08974	1.85E-08	0.0025	0.0068	0.7133	-0.0095	0.0082	0.2434	0.0168	0.0124	0.18
rs7823055	T	G	8	-1.8414	0.09437	1.17E-91	0.0019	0.0068	0.779701	-0.0019	0.0081	0.8118	-0.0036	0.0123	0.77
rs7848973	G	A	9	-0.555	0.08441	4.12E-11	0	0.0064	0.9998	0.0092	0.0076	0.2264	-0.0138	0.0116	0.23
rs7849585	T	G	9	-0.6219	0.09684	9.94E-11	0.018	0.0067	0.006845	0.0204	0.008	0.01048	0.02	0.0121	0.1
rs7858672	G	T	9	0.52391	0.08892	3.36E-09	0.0096	0.0068	0.1569	0.0114	0.0081	0.1571	0.0052	0.0122	0.67

rs7866443	A	C	9	-0.6227	0.1092	8.72E-09	0.0153	0.0073	0.0378	0.0212	0.0088	0.01542	0.0051	0.0134	0.7
rs7869617	C	A	9	0.47965	0.08421	1.13E-08	5.00E-04	0.0068	0.9377	5.00E-04	0.0081	0.9507	-0.0083	0.0122	0.5
rs7904993	C	T	10	1.36226	0.1168	4.47E-34	-0.0092	0.0098	0.3453	-0.0224	0.0117	0.05662	0.0105	0.0178	0.56
rs7907903	T	G	10	-0.5994	0.08663	3.77E-12	-0.01	0.0073	0.1678	-0.0142	0.0087	0.1026	-0.0118	0.013	0.37
rs7909838	A	C	10	-0.6262	0.08671	3.91E-13	0.0028	0.0065	0.668399	0.0054	0.0077	0.4839	-3.00E-04	0.0118	0.98
rs7985861	A	C	13	-0.6062	0.08811	4.71E-12	0.0091	0.0065	0.1622	0.0095	0.0078	0.2248	0.0057	0.0119	0.63
rs7998196	G	T	13	-0.657	0.09026	2.42E-13	0.0009	0.0071	0.9006	0.0016	0.0085	0.8509	-0.0048	0.0129	0.71
rs8007859	T	G	14	0.54214	0.08611	2.65E-10	0.0032	0.0065	0.6243	0.001	0.0078	0.9002	0.004	0.0118	0.73
rs8019424	G	T	14	0.61804	0.08528	3.39E-13	-0.001	0.0069	0.887	0.0052	0.0083	0.5302	-0.0051	0.0124	0.68
rs8026464	C	A	15	0.64512	0.09177	1.51E-12	-0.0102	0.0063	0.1036	-0.0098	0.0074	0.1885	-0.0113	0.0114	0.32
rs8043171	T	G	15	-0.704	0.1261	1.53E-08	-0.0049	0.0092	0.5925	-0.0088	0.0109	0.4223	0.005	0.0166	0.76
rs8062459	G	A	16	2.82197	0.2692	7.65E-43	0.0068	0.0248	0.783401	0.0049	0.0293	0.8682	-0.0821	0.047	0.08
rs8076927	G	T	17	0.47257	0.08544	2.94E-08	-0.0121	0.0064	0.05658	-0.0066	0.0076	0.3872	-0.0188	0.0117	0.11
rs8095811	G	T	18	-2.1253	0.2471	6.04E-24	-0.0196	0.0093	0.03446	-0.0245	0.0111	0.02698	-0.0294	0.017	0.08
rs8183334	G	T	20	1.93448	0.09559	3.03E-99	-0.0057	0.0066	0.3869	-0.0075	0.0079	0.3415	-0.0134	0.012	0.26
rs825770	G	T	5	0.59911	0.1021	3.38E-09	-0.0033	0.0074	0.6568	-0.0082	0.0089	0.3523	0.0061	0.0134	0.65
rs886856	G	T	20	-0.5676	0.09254	7.01E-10	0.0074	0.007	0.2925	0.0078	0.0083	0.3502	-7.00E-04	0.0127	0.95
rs895649	G	A	11	1.18111	0.0962	3.96E-36	-0.0131	0.0064	0.04158	-0.0119	0.0077	0.1223	-0.0132	0.0117	0.26
rs9289086	C	A	3	0.65959	0.09295	8.94E-13	0.0059	0.0078	0.4507	0.0078	0.0092	0.4015	0.0376	0.014	0.01
rs9290550	T	G	3	-0.6035	0.09614	2.65E-10	0.0011	0.0069	0.8796	0.0127	0.0083	0.1247	0.001	0.0126	0.94
rs9294379	A	C	6	2.1698	0.1138	8.12E-96	-0.0017	0.0063	0.781101	-0.0029	0.0075	0.6937	0.0109	0.0114	0.34
rs9297398	C	A	8	-2.0174	0.1195	1.45E-75	-0.0032	0.0071	0.6534	0.0058	0.0085	0.4917	-0.0101	0.0129	0.43
rs929958	A	C	4	-0.4745	0.08412	1.56E-08	0.0052	0.0068	0.4419	0.0077	0.0081	0.3413	-0.0054	0.0121	0.66
rs9325969	T	G	22	-0.5015	0.08487	3.12E-09	-6.00E-04	0.0067	0.931	-6.00E-04	0.008	0.9389	0.0021	0.0121	0.87
rs9344740	T	G	6	-0.5817	0.08668	1.60E-11	-0.0029	0.0068	0.6631	-0.0017	0.0081	0.8367	-2.00E-04	0.0123	0.99
rs9350030	G	T	6	0.60432	0.09255	5.19E-11	0.0132	0.0075	0.07934	0.0141	0.0089	0.1156	0.0248	0.0136	0.07
rs935990	C	A	11	0.73376	0.09027	2.56E-16	-0.0129	0.0081	0.1098	-0.0182	0.0098	0.0638	-0.0278	0.0146	0.06
rs9386492	A	C	6	-0.4816	0.08765	3.58E-08	-5.10E-03	0.0067	0.4448	-5.00E-04	0.008	0.9509	-0.0043	0.0121	0.72
rs9406232	A	C	6	-0.5292	0.08864	2.06E-09	0.0114	0.0065	0.079161	0.0131	0.0077	0.089271	0.0093	0.0118	0.43
rs9464641	G	T	6	2.08989	0.1644	2.32E-47	0.0043	0.0153	0.776599	0.0089	0.0182	0.624499	0.023	0.0276	0.41
rs948018	A	C	11	0.52189	0.09472	3.11E-08	-0.0046	0.0068	0.4963	-0.0041	0.0081	0.614301	-0.0011	0.0124	0.93
rs950275	A	C	10	-0.8694	0.09534	2.65E-20	0.0156	0.0078	0.04371	0.0138	0.0093	0.1374	0.0345	0.0139	0.01

rs9514535	A	C	13	-0.5293	0.08628	7.44E-10	-0.0035	0.0065	0.5909	-0.0077	0.0078	0.3225	0.0083	0.0119	0.48
rs9516234	C	A	13	0.6545	0.09342	1.73E-12	0.0219	0.0068	0.001224	0.0209	0.0081	0.00981	0.0203	0.0123	0.1
rs9535343	T	G	13	-0.5192	0.08695	2.06E-09	0.0005	0.0063	0.9404	0.0032	0.0076	0.67	-0.0216	0.0116	0.06
rs9537154	A	C	13	-0.5786	0.1041	2.20E-08	0.0143	0.0071	0.0433	0.0162	0.0084	0.055	0.0175	0.0128	0.17
rs9547491	G	T	13	0.54369	0.0897	1.15E-09	-0.0029	0.0069	0.674099	-0.0069	0.0082	0.3985	8.00E-04	0.0124	0.95
rs9558058	T	G	13	-2.6507	0.2053	4.13E-58	-0.0056	0.0086	0.514801	-0.005	0.0103	0.626	-0.0214	0.0159	0.18
rs9578483	A	C	13	-0.8723	0.1218	2.78E-13	-0.0029	0.008	0.714	0.0025	0.0095	0.793201	-0.0041	0.0146	0.78
rs9655048	A	C	7	-0.594	0.08777	1.05E-11	-0.0103	0.0068	0.1305	-0.0121	0.0081	0.1358	-0.0145	0.0125	0.25
rs971732	C	A	2	-2.1388	0.1195	1.06E-85	0	0.0065	0.9957	0.0024	0.0077	0.755	0.0117	0.0118	0.32
rs9824256	C	A	3	0.5075	0.08745	5.78E-09	0.0059	0.0069	0.3918	0.0118	0.0082	0.1527	0.0085	0.0124	0.49
rs9867503	C	A	3	0.64131	0.0916	1.85E-12	0.0149	0.0068	0.02784	0.0133	0.0081	0.0998	0.0314	0.0123	0.01
rs9869760	A	C	3	-2.0526	0.1062	8.74E-95	0.0034	0.0063	0.585	0.0077	0.0074	0.2987	-0.0046	0.0115	0.69
rs9889777	C	A	17	0.51483	0.09111	1.40E-08	-3.10E-03	0.0064	0.6278	-8.00E-04	0.0076	0.9128	-0.0107	0.0116	0.36
rs9988716	A	C	10	-0.6737	0.113	1.64E-09	0.0074	0.0084	0.3749	0.0049	0.01	0.6215	0.0111	0.0151	0.46

Supplementary Table 2. Detailed information of the selected SNPs between Breast cancer (exposure, N=228951), ER (+) Breast cancer (exposure, N=175475), ER (-) Breast cancer (exposure, N=127442) and Thyroid cancer (Outcome, N=1187).

Exposure	Outcome	SNPs	Effect allele	Other allele	Chr	β for exposure	β for outcome	SE for exposure	SE for outcome	p value for exposure	p value for outcome
Breast cancer	Thyroid cancer	rs10022462	T	C	4	0.0375	-0.0918	0.0062	0.0833	1.55E-09	0.2708
Breast cancer	Thyroid cancer	rs10096351	G	A	8	0.1055	0.0038	0.0062	0.0847	1.97E-64	0.964
Breast cancer	Thyroid cancer	rs10885405	T	C	10	0.0465	-0.1363	0.0062	0.0833	5.44E-14	0.1015
Breast cancer	Thyroid cancer	rs11205303	C	T	1	0.0497	0.1579	0.0064	0.0844	1.14E-14	0.06146
Breast cancer	Thyroid cancer	rs1121948	G	A	8	0.0575	0.0898	0.0077	0.0912	7.48E-14	0.3236
Breast cancer	Thyroid cancer	rs11249433	G	A	1	0.0988	0.0917	0.0065	0.0841	1.76E-52	0.2734
Breast cancer	Thyroid cancer	rs11672660	T	C	19	0.0472	-0.1782	0.008	0.1065	4.13E-09	0.09413
Breast cancer	Thyroid cancer	rs11684853	T	G	2	-0.0442	-0.1345	0.0062	0.0841	1.12E-12	0.1098
Breast cancer	Thyroid cancer	rs11977670	A	G	7	0.0522	-0.0049	0.0063	0.0835	1.05E-16	0.9529
Breast cancer	Thyroid cancer	rs12250948	C	T	10	-0.0576	-0.0315	0.0074	0.0988	1.04E-14	0.748001
Breast cancer	Thyroid cancer	rs12479355	G	A	2	-0.0426	0.0315	0.0076	0.0974	2.36E-08	0.7467
Breast cancer	Thyroid cancer	rs12487185	G	A	3	0.045	0.1363	0.0066	0.094	1.21E-11	0.1478
Breast cancer	Thyroid cancer	rs1268974	G	A	10	-0.0793	0.1182	0.0063	0.0852	3.71E-36	0.1649
Breast cancer	Thyroid cancer	rs12990503	G	C	2	-0.073	-0.3914	0.007	0.0905	9.99E-26	1.44E-05
Breast cancer	Thyroid cancer	rs1707302	G	A	1	0.0364	0.0802	0.0066	0.091	2.95E-08	0.3781
Breast cancer	Thyroid cancer	rs170801	A	C	18	-0.0591	-0.0385	0.007	0.0948	3.17E-17	0.6844
Breast cancer	Thyroid cancer	rs17156577	C	T	7	0.0578	-0.1253	0.0098	0.1144	4.25E-09	0.2734
Breast cancer	Thyroid cancer	rs17185310	A	G	1	0.0395	0.1372	0.0069	0.0899	8.70E-09	0.1276
Breast cancer	Thyroid cancer	rs17268829	C	T	7	0.0495	0.1848	0.0068	0.0955	4.49E-13	0.052849
Breast cancer	Thyroid cancer	rs2016394	A	G	2	-0.0425	-0.0262	0.0062	0.0852	6.23E-12	0.758399
Breast cancer	Thyroid cancer	rs2253012	T	C	14	0.0433	0.0751	0.0065	0.0844	3.13E-11	0.3731
Breast cancer	Thyroid cancer	rs2432539	G	A	16	-0.0349	-0.0363	0.0064	0.084	4.02E-08	0.668599
Breast cancer	Thyroid cancer	rs2506889	T	C	1	-0.0615	-0.0447	0.0067	0.0919	2.38E-20	0.6269
Breast cancer	Thyroid cancer	rs2747652	C	T	6	0.0663	-0.1103	0.0062	0.0834	1.31E-26	0.1861
Breast cancer	Thyroid cancer	rs2787486	C	A	17	-0.0758	0.1204	0.0068	0.091	5.56E-29	0.1852
Breast cancer	Thyroid cancer	rs2965183	A	G	19	0.0445	-0.0151	0.0065	0.0882	6.31E-12	0.8642
Breast cancer	Thyroid cancer	rs35910339	G	C	9	0.0462	-0.1587	0.0067	0.0899	5.41E-12	0.076871

Breast cancer	Thyroid cancer	rs3903072	T	G	11	-0.0434	0.0432	0.0062	0.0836	2.25E-12	0.6048
Breast cancer	Thyroid cancer	rs4081859	A	G	5	0.0521	-0.009	0.0073	0.1005	7.08E-13	0.9254
Breast cancer	Thyroid cancer	rs418053	C	G	6	-0.0465	0.0782	0.0063	0.0836	1.20E-13	0.3494
Breast cancer	Thyroid cancer	rs4496150	A	C	16	-0.0416	0.1714	0.0072	0.0896	8.09E-09	0.05581
Breast cancer	Thyroid cancer	rs4702131	C	T	5	-0.0424	0.0461	0.0062	0.0832	1.00E-11	0.579599
Breast cancer	Thyroid cancer	rs4820318	A	G	22	-0.0475	-0.0144	0.0064	0.0872	1.03E-13	0.8686
Breast cancer	Thyroid cancer	rs4848599	C	T	2	0.0933	-0.098	0.01	0.1236	1.58E-20	0.4267
Breast cancer	Thyroid cancer	rs4971059	A	G	1	0.0424	-0.0328	0.0064	0.0969	4.83E-11	0.735099
Breast cancer	Thyroid cancer	rs527616	G	C	18	0.0499	-0.0478	0.0064	0.0867	6.70E-15	0.580399
Breast cancer	Thyroid cancer	rs532436	A	G	9	0.0431	-0.2322	0.0078	0.1024	2.91E-08	0.0232
Breast cancer	Thyroid cancer	rs56387622	C	T	3	-0.0942	-0.0905	0.0106	0.1239	5.47E-19	0.4653
Breast cancer	Thyroid cancer	rs58058861	A	G	3	0.0474	-0.0418	0.0074	0.103	1.91E-10	0.685199
Breast cancer	Thyroid cancer	rs62048402	A	G	16	-0.0624	-0.1913	0.0063	0.0841	3.50E-23	0.02289
Breast cancer	Thyroid cancer	rs630965	T	C	9	0.0992	0.169	0.0064	0.0863	3.21E-54	0.05002
Breast cancer	Thyroid cancer	rs6472903	T	G	8	0.0778	0.1378	0.0083	0.1198	4.43E-21	0.2496
Breast cancer	Thyroid cancer	rs6562760	G	A	13	0.0443	0.0754	0.0073	0.0935	1.49E-09	0.4197
Breast cancer	Thyroid cancer	rs6569648	T	C	6	0.0512	-0.009	0.0073	0.0977	2.98E-12	0.9233
Breast cancer	Thyroid cancer	rs6596100	T	C	5	-0.0439	-0.0327	0.0076	0.0992	7.74E-09	0.7417
Breast cancer	Thyroid cancer	rs6597981	G	A	11	0.0439	-0.0533	0.0062	0.0841	1.35E-12	0.526
Breast cancer	Thyroid cancer	rs6787391	T	C	3	0.0567	-0.1478	0.0064	0.0953	9.07E-19	0.1208
Breast cancer	Thyroid cancer	rs6815814	C	A	4	0.052	0.0497	0.0072	0.0843	6.13E-13	0.5562
Breast cancer	Thyroid cancer	rs7072776	G	A	10	-0.0618	-0.0227	0.0068	0.0872	1.75E-19	0.7915
Breast cancer	Thyroid cancer	rs7223535	A	G	17	-0.0439	-0.0364	0.007	0.0945	3.53E-10	0.7004
Breast cancer	Thyroid cancer	rs7258465	C	T	19	-0.0725	-0.0147	0.0066	0.0906	2.79E-28	0.8711
Breast cancer	Thyroid cancer	rs72725173	A	G	14	0.0559	-0.0225	0.0085	0.0923	3.88E-11	0.808
Breast cancer	Thyroid cancer	rs7626742	T	G	3	0.1046	-0.0677	0.0062	0.0845	1.61E-63	0.4263
Breast cancer	Thyroid cancer	rs7697216	C	T	4	0.1039	0.2764	0.0098	0.1351	1.61E-26	0.040301
Breast cancer	Thyroid cancer	rs77554484	A	C	15	-0.0741	0.0354	0.0093	0.1208	1.28E-15	0.767701
Breast cancer	Thyroid cancer	rs78440108	T	C	14	-0.0595	-0.1141	0.0084	0.1042	1.62E-12	0.2734
Breast cancer	Thyroid cancer	rs7904249	A	G	10	0.0764	0.0344	0.0084	0.1124	6.98E-20	0.760901
Breast cancer	Thyroid cancer	rs7971	G	A	7	-0.0365	-0.0195	0.0065	0.0857	1.93E-08	0.8204
Breast cancer	Thyroid cancer	rs9284657	G	C	4	0.0366	0.0836	0.0062	0.0835	3.36E-09	0.3165

Breast cancer	Thyroid cancer	rs9361840	G	A	6	0.0541	0.1536	0.0072	0.0998	7.14E-14	0.1247
Breast cancer	Thyroid cancer	rs941764	G	A	14	0.0463	0.0149	0.0065	0.0887	8.21E-13	0.8653
Breast cancer	Thyroid cancer	rs9693444	C	A	8	-0.0626	0.0212	0.0066	0.0854	1.60E-21	0.8032
Breast cancer	Thyroid cancer	rs9833888	T	G	3	0.0457	0.001	0.0074	0.1032	5.15E-10	0.9942
Breast cancer	Thyroid cancer	rs9952980	C	T	18	-0.0478	-0.0527	0.0065	0.0876	1.65E-13	0.5477
ER (+)	Thyroid cancer	rs10423928	A	T	19	0.0557	-0.1183	0.0095	0.1088	5.09E-09	0.2763
ER (+)	Thyroid cancer	rs10828249	A	G	10	0.0726	0.1026	0.0077	0.0842	6.72E-21	0.2232
ER (+)	Thyroid cancer	rs10896047	A	G	11	-0.0493	0.0432	0.0074	0.0836	2.32E-11	0.6048
ER (+)	Thyroid cancer	rs11133838	G	A	5	-0.0415	0.0456	0.0075	0.0833	2.83E-08	0.5835
ER (+)	Thyroid cancer	rs11205277	G	A	1	0.0562	0.1579	0.0075	0.0844	8.38E-14	0.06146
ER (+)	Thyroid cancer	rs11249433	G	A	1	0.123	0.0917	0.0077	0.0841	4.73E-58	0.2734
ER (+)	Thyroid cancer	rs12110303	T	G	5	-0.074	0.0325	0.0107	0.1128	4.80E-12	0.772999
ER (+)	Thyroid cancer	rs12481286	T	G	20	0.0507	0.0545	0.0091	0.0945	2.79E-08	0.5611
ER (+)	Thyroid cancer	rs12703654	A	C	7	0.0587	-0.0049	0.0077	0.0835	3.10E-14	0.9529
ER (+)	Thyroid cancer	rs12711947	C	T	2	0.0864	-0.098	0.0124	0.1236	3.41E-12	0.4267
ER (+)	Thyroid cancer	rs16857609	T	C	2	0.0721	0.2738	0.0083	0.0879	3.43E-18	0.001792
ER (+)	Thyroid cancer	rs16867692	G	A	8	0.0576	-0.0775	0.0096	0.1136	2.10E-09	0.4947
ER (+)	Thyroid cancer	rs17035305	A	C	1	-0.0436	-0.0648	0.0079	0.0921	4.15E-08	0.4821
ER (+)	Thyroid cancer	rs1707302	G	A	1	0.0466	0.0802	0.0078	0.091	2.75E-09	0.3781
ER (+)	Thyroid cancer	rs170801	A	C	18	-0.0794	-0.0385	0.0084	0.0948	2.99E-21	0.6844
ER (+)	Thyroid cancer	rs17268829	C	T	7	0.0517	0.1848	0.0082	0.0955	2.40E-10	0.052849
ER (+)	Thyroid cancer	rs17617028	A	G	4	0.0569	-0.0281	0.0087	0.1021	7.64E-11	0.783
ER (+)	Thyroid cancer	rs17838698	T	C	3	0.0624	0.1756	0.008	0.0914	5.78E-15	0.05438
ER (+)	Thyroid cancer	rs1812715	G	A	17	0.0908	-0.1258	0.0081	0.0938	2.66E-29	0.1804
ER (+)	Thyroid cancer	rs2016394	A	G	2	-0.0603	-0.0262	0.0074	0.0852	3.22E-16	0.758399
ER (+)	Thyroid cancer	rs2253012	T	C	14	0.0511	0.0751	0.0078	0.0844	5.19E-11	0.3731
ER (+)	Thyroid cancer	rs28649231	A	G	14	0.0651	-0.0099	0.0095	0.094	8.80E-12	0.9159
ER (+)	Thyroid cancer	rs2957449	C	T	8	0.0475	0.1436	0.0076	0.0859	5.24E-10	0.094291
ER (+)	Thyroid cancer	rs310302	A	G	8	0.0444	-0.0554	0.0075	0.0858	3.63E-09	0.5183
ER (+)	Thyroid cancer	rs3217992	T	C	9	-0.0576	-0.0456	0.0076	0.0834	4.60E-14	0.584201
ER (+)	Thyroid cancer	rs35273427	C	T	1	0.0937	0.1124	0.016	0.1866	4.87E-09	0.5462
ER (+)	Thyroid cancer	rs418053	C	G	6	-0.0524	0.0782	0.0075	0.0836	2.28E-12	0.3494

ER (+)	Thyroid cancer	rs4703870	A	G	5	0.0613	-0.009	0.0088	0.1005	3.32E-12	0.9254
ER (+)	Thyroid cancer	rs4820318	A	G	22	-0.0632	-0.0144	0.0076	0.0872	1.10E-16	0.8686
ER (+)	Thyroid cancer	rs4894562	A	G	3	0.0519	-0.0418	0.0089	0.103	4.57E-09	0.685199
ER (+)	Thyroid cancer	rs490706	A	G	3	0.1148	-0.062	0.0074	0.0844	3.28E-54	0.4594
ER (+)	Thyroid cancer	rs4971059	A	G	1	0.0519	-0.0328	0.0077	0.0969	1.27E-11	0.735099
ER (+)	Thyroid cancer	rs4980029	G	A	10	0.0759	0.0344	0.0098	0.1124	1.24E-14	0.760901
ER (+)	Thyroid cancer	rs527616	G	C	18	0.0515	-0.0478	0.0076	0.0867	1.37E-11	0.580399
ER (+)	Thyroid cancer	rs61988429	T	C	14	-0.0486	-0.141	0.0087	0.1034	2.52E-08	0.1726
ER (+)	Thyroid cancer	rs62048402	A	G	16	-0.0593	-0.1913	0.0075	0.0841	2.16E-15	0.02289
ER (+)	Thyroid cancer	rs6472903	T	G	8	0.0784	0.1378	0.0099	0.1198	2.01E-15	0.2496
ER (+)	Thyroid cancer	rs6762558	G	A	3	0.0618	-0.1397	0.0076	0.0945	3.18E-16	0.1394
ER (+)	Thyroid cancer	rs6815814	C	A	4	0.055	0.0497	0.0086	0.0843	1.59E-10	0.5562
ER (+)	Thyroid cancer	rs7153397	T	C	14	0.0536	0.0875	0.0081	0.0868	3.36E-11	0.3129
ER (+)	Thyroid cancer	rs7223535	A	G	17	-0.0463	-0.0364	0.0084	0.0945	3.20E-08	0.7004
ER (+)	Thyroid cancer	rs7280197	C	T	21	0.0461	0.0797	0.0082	0.0864	1.70E-08	0.3573
ER (+)	Thyroid cancer	rs7463114	C	T	8	-0.0684	0.0212	0.0078	0.0854	2.44E-18	0.8032
ER (+)	Thyroid cancer	rs7697216	C	T	4	0.1338	0.2764	0.0118	0.1351	6.06E-30	0.040301
ER (+)	Thyroid cancer	rs7729559	T	C	5	0.057	0.0119	0.0088	0.09	9.31E-11	0.898
ER (+)	Thyroid cancer	rs78029804	T	C	15	-0.076	0.0583	0.011	0.1239	4.57E-12	0.6391
ER (+)	Thyroid cancer	rs811458	C	T	9	0.0511	-0.1587	0.008	0.0899	1.72E-10	0.076871
ER (+)	Thyroid cancer	rs9952980	C	T	18	-0.0549	-0.0527	0.0078	0.0876	1.48E-12	0.5477
ER (-)	Thyroid cancer	rs10069690	T	C	5	0.1613	0.2311	0.013	0.0935	1.54E-35	0.01323
ER (-)	Thyroid cancer	rs10096351	G	A	8	0.07	0.0038	0.0114	0.0847	7.14E-10	0.964
ER (-)	Thyroid cancer	rs10885405	T	C	10	0.0716	-0.1363	0.0113	0.0833	2.00E-10	0.1015
ER (-)	Thyroid cancer	rs12129456	G	T	1	-0.0835	-0.1075	0.0118	0.0875	1.41E-12	0.2191
ER (-)	Thyroid cancer	rs12990503	G	C	2	-0.0734	-0.3914	0.0126	0.0905	6.48E-09	1.44E-05
ER (-)	Thyroid cancer	rs17828955	C	T	14	-0.0797	-0.1433	0.013	0.1142	7.76E-10	0.2095
ER (-)	Thyroid cancer	rs183438976	T	C	22	0.1285	0.062	0.0186	0.1488	4.51E-12	0.6747
ER (-)	Thyroid cancer	rs2169137	C	G	1	-0.128	0.0042	0.0128	0.0926	1.42E-23	0.9638
ER (-)	Thyroid cancer	rs2747652	C	T	6	0.0991	-0.1103	0.0113	0.0834	1.93E-18	0.1861
ER (-)	Thyroid cancer	rs3769823	G	A	2	-0.0684	-0.1476	0.0121	0.0877	1.76E-08	0.09251
ER (-)	Thyroid cancer	rs4143044	G	T	13	0.0833	0.0754	0.0135	0.0935	6.89E-10	0.4197

ER (-)	Thyroid cancer	rs55872725	T	C	16	-0.0784	-0.1916	0.0115	0.0841	8.59E-12	0.02254
ER (-)	Thyroid cancer	rs56069439	A	C	19	0.155	-0.0868	0.0121	0.0956	1.25E-37	0.3644
ER (-)	Thyroid cancer	rs56309329	T	A	6	0.0734	0.1536	0.0131	0.0985	2.10E-08	0.1187
ER (-)	Thyroid cancer	rs56687477	A	G	8	-0.1114	-0.0153	0.016	0.0974	3.19E-12	0.8754
ER (-)	Thyroid cancer	rs616402	T	C	1	-0.1172	0.0373	0.0124	0.0937	2.65E-21	0.6934
ER (-)	Thyroid cancer	rs6569648	T	C	6	0.0741	-0.009	0.0135	0.0977	4.26E-08	0.9233
ER (-)	Thyroid cancer	rs7710996	G	A	5	-0.0773	0.0643	0.0132	0.1063	5.19E-09	0.5451
ER (-)	Thyroid cancer	rs9397437	A	G	6	0.2733	0.0825	0.0206	0.1444	2.76E-40	0.5682
